# Looking Through the COVID-19 Window of Opportunity: Future Scenarios Arising From the COVID-19 Pandemic Across Five Case Study Sites

**DOI:** 10.3389/fpsyg.2021.635686

**Published:** 2021-07-07

**Authors:** Isabell Richter, Arlene Avillanosa, Victoria Cheung, Hong Ching Goh, Sofia Johari, Susan Kay, Carya Maharja, Thu Hà Nguyễn, Sabine Pahl, Jito Sugardjito, Joel Sumeldan, Quyen van Nguyen, Hien Thuc Vu, Wan Nur Syazana Wan Mohamad Ariffin, Melanie C. Austen

**Affiliations:** ^1^School of Psychology, University of Plymouth, Plymouth, United Kingdom; ^2^Psykologisk Institutt, Norwegian University for Science and Technology, Trondheim, Norway; ^3^College of Fisheries and Aquatic Sciences, Western Philippines University-Puerto Princesa Campus, Puerto Princesa, Philippines; ^4^School of Biological and Marine Sciences, University of Plymouth, Plymouth, United Kingdom; ^5^Department of Urban and Regional Planning, Faculty of Built Environment, University of Malaya, Kuala Lumpur, Malaysia; ^6^Plymouth Marine Laboratory, Plymouth, United Kingdom; ^7^Centre for Sustainable Energy and Resources Management, Universitas Nasional, Jakarta, Indonesia; ^8^Faculty of Social Work, Hanoi National University of Education, Hanoi, Vietnam; ^9^Urban and Environmental Psychology Group, University of Vienna, Vienna, Austria; ^10^Faculty of Biology, Hanoi National University of Education, Hanoi, Vietnam; ^11^Vietnam Man and Biosphere Program National Committee, Vietnam National Commission for UNESCO, Hanoi, Vietnam

**Keywords:** scenarios, window of opportunity, COVID-19, sustainable development, Southeast Asia, coastal communities

## Abstract

The COVID-19 pandemic has caused (and continues to cause) severe disruption in global and local economies and has forced countries, societies, and individuals to adapt quickly to the unprecedented and unpredictable situations. Despite the obvious negative consequences of the pandemic, many have called for efforts to identify transformative opportunities for sustainable development throughout this disorderly time. In the present paper, we explore such potential opportunities in the context of an interdisciplinary, international research project, which is focusing on sustainable marine management in biosphere reserves and marine parks in Southeast Asia. During a virtual workshop conducted as part of the GCRF (Government’s Global Challenges Research Fund) Blue Communities Project, future scenarios were developed depicting the potential effects of the COVID-19 pandemic on five case study sites. All of these sites are in areas of internationally recognized outstanding ecological value (Taka Bonerate Kepulauan-Selayar Biosphere Reserve, Indonesia; Tun Mustapha Park, Sabah, Malaysia; Palawan Biosphere Reserve, Philippines; North Devon Biosphere Reserve, United Kingdom; Cu Lao Cham-Hoi An Biosphere Reserve, Vietnam). At the macro-level, economies, governance structures, and societal norms are undergoing big changes. At the micro-level, the livelihoods, lifestyles, and backyards of local residents have to adapt. Collaboratively, we explored how COVID-19 posed challenges in our five case study sites, but we also focused on the potential COVID-19-related windows of opportunity for future sustainable development. Opportunities could be identified in all three pillars of sustainable development: the environment, the society, and the economy. Although remarkable similarities can be found across all five sites, we conclude that there cannot be a “one-size-fits-all” solution to turn the tide toward achieving sustainable development. Just as before the pandemic, sustainable development starts with engaging with and understanding local environments, challenges, and situations; building on local knowledge; and developing tailor-made solutions for the communities *in situ*.

## Introduction

### Windows of Opportunity

It has been argued that external events, such as natural disasters, or significant personal life changes, such as becoming a parent or moving house, provide windows of opportunity (Wood et al., 2005; Thogersen, 2012; Thomas et al., 2016) in which old habits can be discontinued and new habits can be established more easily (Verplanken et al., 2008). These windows can act as catalysts to change individual behaviors, such as consumption patterns (Schäfer et al., 2012), traffic mode choice (Thomas et al., 2016; Schmidt et al., 2021), and other environmentally relevant behaviors (Verplanken and Roy, 2016). Along the same lines, societies and organizational structures can be transformed by major disasters facilitating new laws to be established or peace processes to be set in motion as described in Birkmann et al. (2010).

The reason for this phenomenon is that habitual behavior is triggered by external cues, which may change or disappear during or after the disruption. In times when habits are (temporarily) disturbed, people have the opportunity to refocus on their values and intentions, as the drivers of their behavior. On those occasions, people are more sensitive to new information and information that is in line with their personal values (Schäfer et al., 2012; Verplanken and Roy, 2016). Similarly, in times of broader transition, contextual constraints and structures may change and allow for a period of adaptation and change. It is assumed that a window of opportunity can be up to 3 months long, indicating a temporal space for new habits or structures to be established (Verplanken and Roy, 2016).

We argue that COVID-19 might, despite the uncontested negative effects, have opened up such a window of opportunity (Schmidt et al., 2021). With a total of almost 151 million cases and over 3.1 million deaths (World Health Organization [WHO], 2020) across almost every country in the world (Guan et al., 2020), as of May 1, 2021, the latest data from the World Health Organization (WHO) confirm that the COVID-19 global pandemic has not abated yet. The pandemic has necessitated the alteration of many aspects of daily lives across the globe. Many governments introduced border closures and repeated, prolonged periods of lockdown, controlling the movement of people from their local districts, restricting almost all forms of direct physical contact outside the immediate family and closing of schools and other educational settings (Dwivedi et al., 2020). Nevertheless, the global responses toward the pandemic have not been well coordinated, with the policies of various governments being constantly in flux (Imtyaz et al., 2020). Global business activities have also been disrupted, causing what has been commented as a “cross-border economic disaster” (Ibn-Mohammed et al., 2020), with global supply chains, including for critical medical supplies and pharmaceuticals, being affected (Shih, 2020).

In many societies, the early impacts of prolonged periods of restrictions on normal activities have been observed to be wide-ranging. Some have pointed out that the mental health and well-being of many people have been, and will continue to be, adversely affected during this time of pandemic (Groarke et al., 2020; Serafini et al., 2020; Torales et al., 2020). Moreover, the pandemic is also predicted to cause the worsening of structural inequalities found in many societies and exacerbated the social risks of already-vulnerable groups (Blundell et al., 2020; Lambert et al., 2020; Sharma, 2020; United Nations Development Programme [UNDP], 2020; van Dorn et al., 2020).

In some areas, positive change could be observed as well. In recent months, fossil fuel emissions dropped significantly (Muhammad et al., 2020), less noise pollution was recorded in many cities around the world (Basu et al., 2021; Terry et al., 2021), and pressured ecosystems started to recover (Patterson Edward et al., 2021). Furthermore, changes in patterns of individual behavior have been observed (de Haas et al., 2020), and many forms of technological innovation that can improve our quality of life have been accelerating (Brem et al., 2020). These COVID-19-provoked transitions can be used as indicators to where sustainable change processes might be leveraged.

We now have the decision to relapse to our environmentally destructive investment patterns and activities or to “build back better” as stated in a recent document on policy responses on the Coronavirus by the Organisation for Economic Co-Operation and Development (OECD, 2020). So far, the build back better targets are formulated on a non-specific global scale, leaving smaller regions and local governments with the question of implementation. Given the limited time window we have, in which COVID-19 still keeps normality on hold, specific areas for positive sustainable change on a local level urgently need to be identified (Lambert et al., 2020).

The aim of this paper is to identify the challenges and windows of opportunity as well as leverage points for sustainable change processes across five case studies, using a future scenario development approach. All five case studies (Taka Bonerate Kepulauan-Selayar Biosphere Reserve, Indonesia; Tun Mustapha Park, Sabah, Malaysia; Palawan Biosphere Reserve, Philippines; North Devon Biosphere Reserve, United Kingdom; and Cu Lao Cham-Hoi An Biosphere Reserve, Vietnam) are areas of internationally recognized outstanding ecological value and are part of the GCRF (Government’s Global Challenges Research Fund) Blue Communities Project, described below. Four of the case study sites are located in South East Asia, representing areas of particular vulnerability to climate change-related pressures (Eckstein et al., 2019). One case study site is located in the United Kingdom, representing a Western counterpoint and compelling opportunity to discuss similarities and differences across geographical regions and cultures. The cultural, historical, and political differences between the European and the South East Asian case study are being considered in the interpretation and discussed.

### GCRF Blue Communities and the Sustainable Development Goals (SDGs)

GCRF Blue Communities^1^ is a project funded by the UK GCRF, in response to the call for “Growing Research Capability.” Through academic–stakeholder collaborations, community co-creation, and co-delivery, the GCRF Blue Communities Project is supporting the development, implementation, and ongoing management of initiatives that promote the sustainable use of marine resources by multiple users. At the same time, it aims to protect the fragile marine ecosystems and support the livelihoods, food security, health, and well-being of the people in these coastal communities.

Within the GCRF Blue Communities Project, the research capabilities of the team are being increased through training and co-developed research with stakeholders in the case study areas. Interdisciplinary research is being applied to foster health and well-being, and protecting both human and ecosystem health, within the framework of the United Nations’ (UN) SDGs. Particular focus is on the SDGs: SDG 1, no poverty; SDG 2, zero hunger; SDG 3, good health and well-being; and SDG 14, life below water. The UN’s SDGs are an urgent call for action by all countries in a global partnership (UN)^2^. As highlighted by Lambert et al. (2020), the impact of the COVID-19 pandemic goes beyond health and well-being, potentially affecting society in many ways that underpin the UN SDGs. It is therefore key to identify not only the vulnerabilities and threats posed by the COVID-19 pandemic on societies, the environment and economies but also potential opportunities to build back better.

### Scenarios as Way to Communicate and Connect to the Future

The human brain is wired to prioritize immediate futures and short-term time horizons (Gardner and Stern, 1996). This stands in conflict with sustainability as a concept that revolves around the long-term consequences of our current behavior (Pahl et al., 2014). To realize sustainability, we need an active dialogue between today and potential long- and short-term futures.

Future scenarios have been promoted as a way to render concrete links between the future and humans’ present situations or actions, which may benefit the sustainability agenda (Boyko et al., 2012; Arnocky et al., 2014). Scenarios, in this sense, can be described as coherent descriptions of imagined futures deemed to be plausible. However, in most cases, the depicted futures are far away (end-of-century scenarios), on a global scale or in the form of complex biophysical predictions (Sheppard et al., 2011; Intergovernmental Panel on climate Change, 2014). These forms of scenarios are eligible within their discipline, but they do not meet the requirements for effective communication to overcome psychological distance or reactance (Kollmuss and Agyeman, 2002; Nicholson-Cole, 2005; Maibach et al., 2010; Moser and Dilling, 2011). One way to address such shortcomings is to include diverse perspectives in the scenario development and adapt the scenario design to the respective target audiences (Johnson et al., 2012). It has been demonstrated that the incorporation of such diverse inputs is best undertaken using a participatory approach with groups representing different, or sometimes, opposing perspectives (for instance in Hulse et al., 2004; Enfors et al., 2008; Kok et al., 2011).

Using a participatory approach can be useful for the development of more robust, effective, and representative future scenarios. Participatory scenarios can make the future more tangible by painting detailed pictures based on local references. Thereby, participatory scenario development can address a number of barriers such as the overwhelming scale of the problem, uncertainty, scientific abstraction, and the predominantly global nature of the available modeling and scenarios (Sheppard, 2008; Burch, 2010; Pahl et al., 2014). Potential downsides of participatory scenario development that need to be considered are a risk of poor comparability across different groups and the high subjectivity of the method (Kok et al., 2007). We need to be aware that the scenarios developed are based on personal perceptions and expectations and not a definite representation of the real world from a natural science perspective.

Alternative scenarios cannot only bridge the gaps between today and the future, but also between various stakeholders and community members. They have been claimed to be effective tools of communication as they are capable of engaging broad audiences and making complex systems more tangible (Chaudhury et al., 2013; Reed et al., 2013; Nygrén, 2019). Examples of co-created scenarios that have been used for strategic conversation between stakeholder groups, scientists, and policy makers are the UK National Ecosystem Assessment (NEA) Scenarios (Lead et al., 2010). Scenarios like these can help to identify important choices we need to make and further, illustrate the consequences of those choices for ecosystems, society, and the economy. Beyond the product, i.e., the scenarios as deliberate tools for decision making, the process of scenario development itself is valuable. It gives a voice to experts and non-experts and pushes them to think and communicate beyond their own discipline and peer group (Haines-Young et al., 2014).

Within GCRF Blue Communities, alternative future scenarios were developed for each case study site, which helped all involved researchers and stakeholders to familiarize themselves with the locations and to create springboards for sustainable development. However, to take into account the impacts of the unexpected COVID pandemic, these scenarios need to be revisited, and this is the focus of this paper.

## Materials and Methods

### Process

The aim of the virtual workshop held in July 2020 during the GCRF Blue Communities’ third Annual Meeting was to create five future scenarios to establish the potential impacts of the COVID-19 pandemic at each case study site (Taka Bonerate Kepulauan-Selayar Biosphere Reserve, Indonesia; Tun Mustapha Park, Sabah, Malaysia; Palawan Biosphere Reserve, Philippines; North Devon Biosphere Reserve, United Kingdom; and Cu Lao Cham-Hoi An Biosphere Reserve, Vietnam). Each case study team had already, together with their local stakeholders and community members, developed three alternative future scenarios looking approximately 10 years into the future (business-as-usual scenario (BAU), best-case scenario, and worst-case scenario) before the COVID pandemic hit (see Supplementary Table 1). These scenarios served as education and communication tools by GCRF Blue Communities’ researchers, communities, and stakeholders.

The development of the COVID-19 scenarios took place in the form of group discussions held *via* Zoom. Compared to face-to-face interactions and workshops, virtual interactions have a number of advantages and disadvantages in regard to accessibility, equality, and engagement. For a project-related overview of this issue, please refer to Richter et al. (2021). Each case study was represented by researchers from different disciplines (*n* = 3–6 for each case study). The team members have been selected based on a history of close collaboration with the local communities and stakeholders in the case study sites. Directly involving stakeholders and community members has been considered but deemed not feasible due to unequal access to stable internet connections, COVID-19-related movement restrictions, and sensitivity considerations for local populations already experiencing economic and social pressures.

To set the scene, each GCRF Blue Communities case study team revisited their BAU scenario (approximately 5 min allocated). The original scenarios have been narrated from the perspective of a stereotypical local family experiencing future changes in 10 years’ time. This is based on research by Pahl and Bauer (2013) looking at perspective-taking in the context of imagining the future. They found that perspective taking induces higher levels of behavioral intentions and engagement with the topic as compared to focusing on objective facts only. The COVID-19 scenarios are therefore narrated from the same perspective. Each case study team was placed in a breakout group and given 30 min in total to identify the changes and opportunities imposed by the COVID-19 pandemic at their case study site. With the BAU as orientation, the groups were able to identify the parts of the BAU scenarios that changed, positively as well as negatively, through the impact of the pandemic (approximately 15 min allocated). This alternative scenario development drew upon the existing knowledge about the case study site, insights into the current situation as well as continuous community and stakeholder communication that has been undertaken by the case study teams during the project. In the final 10 min of the breakout session, the case study teams identified which of these changes could be turned into opportunities for sustainable development, using the SDG subgoals as orientation. Once the breakout sessions ended, each team shared their COVID-19 scenario discussing similarities and differences between the case study sites. As an overall output, the teams developed five COVID-19 scenarios, representing the projected future of their case study site, approximately 10 years after the pandemic. Please refer to Supplementary Table 1 for the details of the original BAU scenario, the alternative COVID-19 scenarios, and the identified opportunities. In the case of the United Kingdom, no BAU scenario has been co-developed before the pandemic. Instead, the workshop participants developed the Devon Biosphere Reserve BAU (before the workshop) as well as the Devon Biosphere Reserve COVID-19 scenario based on their expert knowledge of the area.

### Materials

Due to positive experiences regarding connectivity, sound, and interactive features, Zoom was chosen as the online platform to connect the researchers across five countries. In two countries (the Philippines and Vietnam), the researchers were able to meet physically in one location, whereas all other participants joined individually from their offices or homes due to the COVID-19 movement restrictions. In terms of materials, each group had the documentation of their previously developed BAU scenario available. Further, the virtual workshop utilized flip charts, pens/markers, post-it notes, and a camera/mobile phone with a camera to facilitate discussions and person-to-person interaction as recommended in the study by Richter et al. (2021). The workshop was moderated by one of the UK-based researchers, documented by the project manager, and video-recorded for later reference.

### Scenarios

The materials produced in the workshop are the session recording and transcript as well as flip charts and post-it notes, which were particularly extensive in the groups that were able to meet in person. These transcripts and notes were collectively synthesized after the workshop, supported by the recording (for a summary, see Supplementary Table 1). Each group developed one case study-specific alternative future scenario for the time approximately 10 years after the COVID-19 pandemic.

#### Palawan, the Philippines

Before COVID-19, agriculture, fisheries, and tourism were the primary economic activities in the province of Palawan. Especially the tourism sector was growing expeditiously and was expected to provide local employment as more tourists visited the island. Palawan has been ranked the number one travel destination by Travel + Leisure magazine in their 2013, 2016, 2017, and 2020 edition.

During the COVID-19 pandemic, the link between tourism and agri-fisheries in Palawan became more evident. Tourism had been creating local income and employment because of the high demand for farm products and seafood. As tourism stands still due to the pandemic, farmers and fishers cannot sell their products and catch. This is aggravated by the restrictions on local travel because the products cannot be transported to the markets in Puerto Princesa or Manila.

Before this pandemic, Palawan’s coastal communities exported fish and seafood to other parts of the country and abroad. The Philippines’s popular and lucrative live reef fish export was mainly sourced from Palawan. The travel ban affected this important economic sector. As income decreases, food and other necessities cannot be bought anymore. Consequently, families that own the small local stores known as “Sari-Sari” lose their income as well.

**FIGURE 1:**
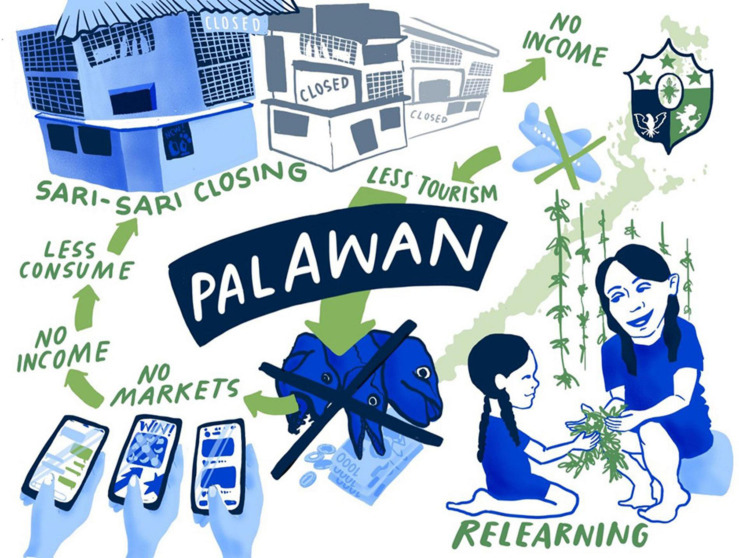
COVID-19 scenario for Palawan. Illustration by Lina Ernst Illustrations.

Amid the unstable health and economic situation brought by the COVID-19 pandemic, we see opportunities for fisheries and agriculture as well. Children who have been studying in the city are staying home. Now, they help with the farming and fishing activities of their families. Through this experience, the young generations of Palaweños can relearn the lost skills and appreciate the importance of agriculture and fisheries for food security. Another opportunity potentially leading to long-term change on the island is that people start to explore selling their goods online. For the environment, the decrease in fishing and farming activities provides a unique chance to recover. For an overview over the key aspects of the COVID-19 scenario for Palawan (see Figure 1).

#### Tun Mustapha Park (TMP), Sabah, Malaysia

Before COVID-19, the rural communities produced agricultural products to sell them in nearby settlements in exchange of other essential products. One of the major disruptions during the movement control order is that rural and marginalized groups are now cut off from their access to essential foods such as rice, sugar, milk or cooking oil, reinforcing pressure on this group with already low income.

Meanwhile, the influx of undocumented people from the Philippines and Indonesia has been a complex issue in Sabah for decades. The migrants have limited access to healthcare and education, as they do not possess the proper documentation. This limits their opportunities for formal employment, which further exacerbates poverty among the communities. In order to sustain their livelihood, some fishermen turn to illegal activities such as poaching or the use of destructive fishing methods. Government agencies argue that they lack the resources to handle the situation.

During the COVID-19 pandemic, the undocumented communities in TMP were heavily impacted by the lockdowns and movement control orders (MCOs). Confusion about the new laws created fear of going out to fish or getting essential supplies such as medicine, fuel, and freshwater. In addition, the limited awareness about how the virus transmits substantially increased the risk of the undocumented communities being hit by COVID-19. Not being able to pay for the hospital charges as well as the fear of being arrested kept the undocumented communities from seeking medical help. These unfortunate circumstances lead to an explosive rise of cases in Sabah, forcing the government to act. This can be interpreted as an opportunity arising from COVID-19: legal frameworks related to undocumented people and their access to basic facilities including education and healthcare services will be revisited and adjusted; social systems will be improved taking into consideration the health and well-being as well as equality of everyone.

**FIGURE 2:**
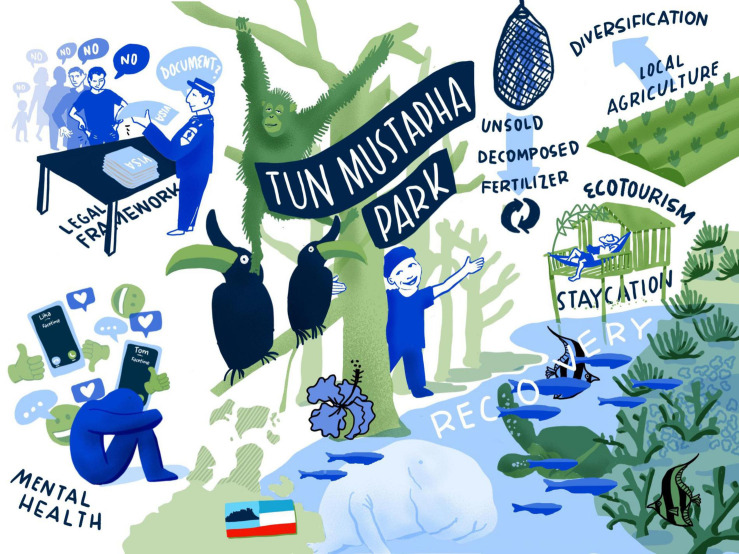
COVID-19 scenario for Tun Mustapha Park. Illustration by Lina Ernst Illustrations.

Another opportunity is the higher levels of appreciation for nature within the population. Spending time in nature provides a remedy for stress and mental health issues. This combined with the reduction in (destructive) fishing activities provides a chance for fish stocks and other marine species to recover. Differences in recovery rates point to where marine protected areas would be appropriately located and offer the opportunity to strengthen measures to protect marine biodiversity for the long term. In addition, the pandemic prompted communities to adopt a more hygienic lifestyle to prevent the spread of the virus. For an overview over the key aspects of the COVID-19 scenario for Tun Mustapha Park (see Figure 2).

#### Cu Lao Cham Islands, Hoi An, Quang Nam, Vietnam

Before the COVID-19 pandemic, Cu Lao Cham was usually busy with tourists and visitors from all over the world. Hundreds of boats and canoes departed every day from the mainland in Hoi An, to bring thousands of people to the island. Tourism has generated many job opportunities and raised local people’s income. The standard livelihoods have shifted from fishing to hospitality services. Homestays, restaurants, shops, and cafeterias have sprung up, and local people consider tourism services such as offering boat rides as a lucrative profession.

As a consequence of the COVID-19 pandemic, the government had imposed a national lockdown and social distancing. Some famous islands in Vietnam, including Cu Lao Cham, had to suspend all tourism operations. This implied that only shipments of goods and necessities for local people on the island were permitted; however, visiting the island for non-essential purposes was declared illegal. These restrictions caused severe consequences for the local population. Many people lost their jobs and income, children had to interrupt their education, and all schools were closed.

**FIGURE 3:**
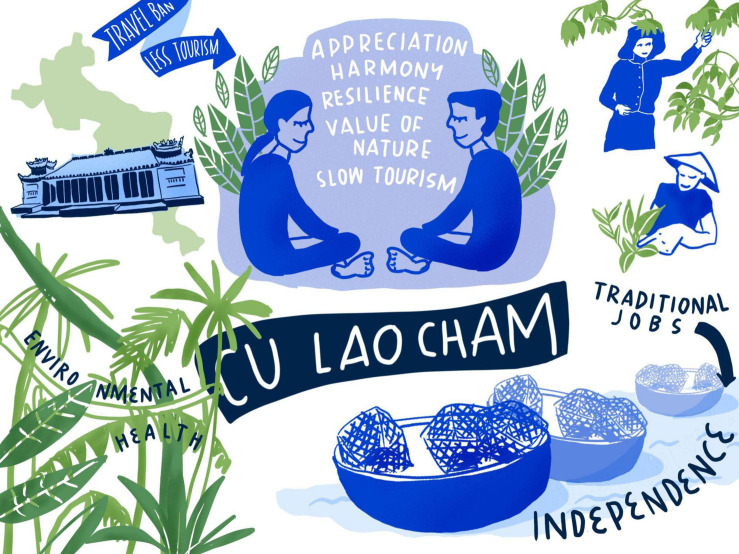
COVID-19 scenario for Cu Lao Cham. Illustration by Lina Ernst Illustrations.

At the same time, people returned to traditional jobs such as fishing, net making, or forest leaf collecting to provide local self-supply. This made them independent from the mainland. Although the local people’s income decreased significantly, they started appreciating the island in a less crowded state. They started living more harmonious and less pressured lifestyles. Traditional local culture experienced a comeback. Their increased value of nature and environmental conservation might provide an opportunity for the rise of slow tourism after the pandemic. This would bring less, but higher-quality tourism to the island, cause less pollution, and help the marine ecosystem in the waters surrounding the island to recover. For an overview of the key aspects of the COVID-19 scenario for Cu Lao Cham (see Figure 3).

#### Taka Bonerate Kepulauan Selayar Biosphere Reserve, Sulawesi, Indonesia

On the island of Selayar, destructive fishing practices are popular and were predicted to increase due to minimal monitoring and law enforcement before the COVID-19 pandemic. Coastal ecosystems, especially the coral reefs, were subject to heavy damage, impacting the livelihoods of fishers and gleaners who were forced to find alternative jobs. Moreover, forests on the island have increasingly been cleared to provide land for agriculture. Poverty, mental health problems, and criminal activities have been on the rise, especially within the male population, which often forced women toward taking on more responsibility.

The pandemic forced the business-as-usual activities to a halt. As a positive result, disturbances to local ecosystems have been significantly reduced, if only in the coastal areas. This provided a time of recovery for marine ecosystems, especially the coral reefs, acting as a “fish-stock replenishment time.” The recovered fish stocks secure the catch for the local fishermen, and open the discussion for the implementation of no-catch zones or seasons on a governmental level. Many people are also learning new agricultural skills, planting staple foods in their backyards, which will be useful to support their economic self-sufficiency after the pandemic.

**FIGURE 4:**
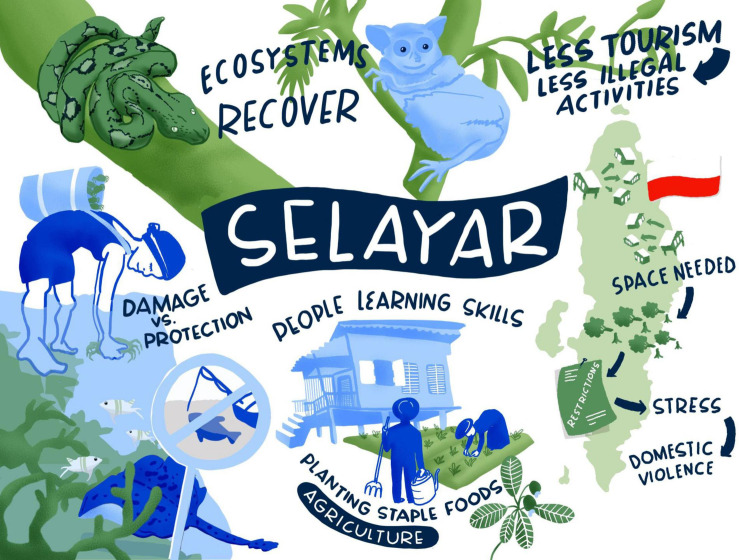
COVID-19 scenario for Selayar. Illustration by Lina Ernst Illustrations.

However, COVID-19 also created challenges especially related to the island becoming more self-sustained and separated from the mainland. The coastal communities will have to intensify gleaning activities, which will cause physical damage to sensitive ecosystems, such as coral reefs. Those who move inland will need more land to build living spaces and plant crops, resulting in deforestation. The restrictions on people’s movements will put further stress on interpersonal relationships giving rise to domestic conflicts and violence. For an overview over the key aspects of the COVID-19 scenario for Selayar (see Figure 4).

#### North Devon Biosphere Reserve, Devon, United Kingdom

The North Devon Biosphere Reserve is a mainly rural area, and its natural beauty and unpolluted environment have attracted visitors from across the United Kingdom and beyond for decades. Before COVID-19, the main economic activities have been tourism and farming, but there was also some office and industrial employment. There have been many small businesses producing speciality products marketed to be of local origin. Restaurants often emphasized their use of local products. Environmental issues have been important to many local residents and tourists. Wages have been below the national average, but employment rates were high. Many households had some form of debt, such as a mortgage or business loan.

As COVID-19 cases started to rise rapidly, a national lockdown in England was imposed during March 2020. All schools and non-essential workplaces were closed for several weeks or months. Tourism stopped completely, so many small businesses lost their income suddenly, without any opportunity to plan or manage implications. Some office workers struggled with working from home due to unstable internet connections in rural areas. Uncertainty and the threat of redundancy put a strain on many, especially those who saw their home at risk if they could not afford rent or mortgage payments. Food banks, which provide emergency help for those in need, saw a rise in demand. With schools closed, parents had to manage childcare and education. In the longer term, the pandemic will cause many businesses to fail, workers will be made redundant, and people will fall into debt.

**FIGURE 5:**
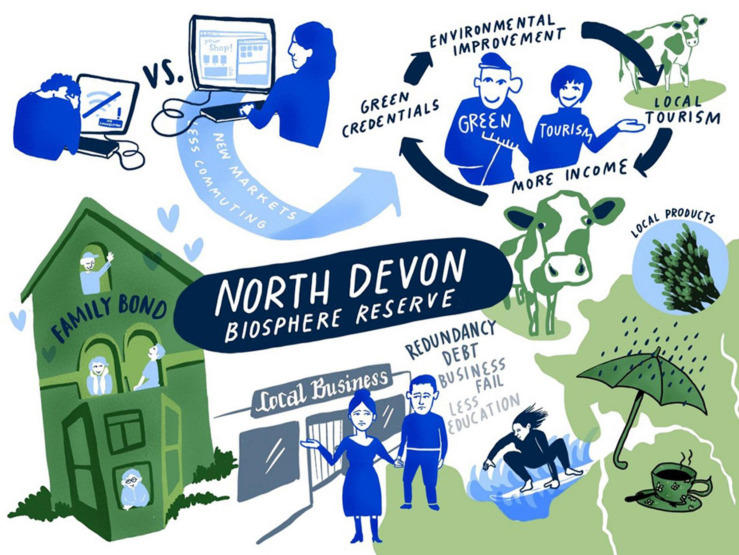
COVID-19 scenario for North Devon Biosphere Reserve. Illustration by Lina Ernst Illustrations.

However, there are opportunities too. Due to travel restrictions imposed by the government, people have been unable or reluctant to travel overseas; thus, domestic UK tourism is likely to increase. In North Devon, this can be marketed as green tourism, avoiding air travel and supporting small-scale local businesses. Money from tourism could be invested in local environmental improvement, in a virtuous cycle where the green credentials attract further tourism. Some business owners have found new markets, often online, and will be able to develop these. There is likely to be a long-term drop in commuting, leading to reductions in local air pollution. The lockdown experience has also changed personal relationships within families as the generations spent more time together, in many cases building a strong foundation for the children. Daily exercise regulations encouraged people to take time to enjoy the natural outdoor spaces, which has been reported to improve health and well-being as well as increase pro-environmental behaviors (Bratman et al., 2019; White et al., 2019; Martin et al., 2020). At times when there were limited provisions available, some people started to home-grow vegetables. For an overview of the key aspects of the COVID-19 scenario for North Devon Biosphere Reserve (see Figure 5).

## Discussion

Each case study site developed a comprehensive scenario describing future developments of how the three pillars of sustainable development, namely, the environment, the society, and the economy, are being shaped by the COVID-19 pandemic. Across the five scenarios, recurring themes have been identified such as the fate of the fisheries industry, the way people procure for their daily needs and health, changes in the ecosystems, and how the environment is perceived as well as the magnified role of the internet in communication activities. In addition, some themes occur that are unique for each of the respective locations.

### Commonalities Across Scenarios

Across all five case study sites, there are several common themes. Economic impacts of the COVID-19 pandemic have been found to be a relevant issue in each case study site. Across scenarios, the restriction of movement and global travel bans had an immediate negative effect on global and local economies (Fotiadis et al., 2021): Many people have lost their source of income, and therefore, especially in the Southeast Asian case studies, struggled to provide the necessities for their families (OECD, 2021). In all five scenarios, this economic blow has forced people to take their focus away from international dependencies and toward local, self-sustained lifestyles, which might be a seminal trend. People are starting to grow their own food, collaborate more within their families and communities, or become tourists in their own country (Erokhin and Gao, 2020; Fan et al., 2021).

Marine and forest ecosystems have had the chance to recover during the national lockdowns, and nature reclaimed some space. Fish stocks are increasing from depleted levels, local water quality is improving, and the extent of litter has reduced due to the break from human intrusion (Patterson Edward et al., 2021). In all five case study sites, some ecosystems have benefitted more than others.

Under the scenarios, together with ecosystem restoration come increased levels of nature appreciation (Pouso et al., 2021). People across all five case studies develop a recent feeling of gratitude for their environment as a space for comfort, pleasure, and social interaction.

Not only in our five case study sites, but certainly around the world, online activities replacing or compensating for face-to-face interactions have been rising abruptly. Almost all areas of life have been transferred to the virtual space in some form, be it for education, news, work, trade, leisure, or social interactions (De’ et al., 2020). So far, this development has mostly been projected to be beneficial (Gabbiadini et al., 2020). It might, however, pose strains on mental health as well as interpersonal and human–nature connections in the future (Kuss et al., 2014; Dong et al., 2020).

### Differences Between Scenarios

Tourism is being discussed as a key theme in all scenarios; however, different trends can be identified between the four South East Asian scenarios and the European scenario. In South East Asia, a region that heavily relies on international tourists, the international travel ban caused the loss of jobs and livelihoods of millions of people, the majority of whom do not have an economic safety net (Fotiadis et al., 2021). Local tourism only accounts for a small percentage of the total revenue and is therefore not able to offset the losses. In the UK scenario, local visitor numbers are rising as people are encouraged to make holidays within their own country and local businesses have the chance to expand. In our UK scenario, the North Devon Biosphere Reserve did not heavily rely on international travel before the pandemic and might therefore benefit from the international travel restrictions. These different trends indicate opposite trends that might emerge in the years after the pandemic. We can’t predict with certainty how global and local tourism will develop after the pandemic, but in the Blue Communities COVID-19 scenarios as well as in the scenarios developed by Wassler and Fan (2021) concerns are being raised about a rebound of tourism during post-COVID-19 (termed as “revenge tourism”). Such “revenge travel” could potentially pose a shock to the environment and the communities. Opportunities for more conscientious and environmentally ethical travel and behavior of both local and international tourists need to be identified, some of which are described in this paper (e.g., slow tourism, eco-tourism).

Dynamics within families have been forced to change during and after the pandemic. Across our case study sites, these changes are perceived to be diverse and again following different trends. In some case studies (North Devon, Palawan), families seem to get closer and interactions and support across generations are increasing which is perceived as positive. In Selayar, the new family dynamics are reported to cause more domestic violence, as family members do not have the opportunity to spend time separately from each other. We assume that both phenomena, increased bonding, and increased violence, will occur across all five case study sites, potentially at varying rates, and cause lasting societal effects in interpersonal interaction (Evans et al., 2020). Observing these phenomena underlines the importance of an inclusive safety net for social and mental health services.

### Unique Themes of Each Case Study Site

Each scenario contains at least one theme that appears to be unique for the particular case study.

In Palawan, this is the live reef food fish trade (LRFFT). For decades prior to the pandemic, about 60–70% of fishing communities were involved in LRFFT (Pomeroy et al., 2008). The economic benefits of this activity have been substantial. However, the unregulated, destructive fishing practices caused the radical decline of fish stocks and reef habitats. The ongoing pandemic has negatively affected the economic gains but has also provided an opportunity for the marine ecosystems to recover. In 10 years’ time, LRFFT in Palawan will be back to the levels from before the pandemic. However, the Philippine government will also invest more funds in fisheries and aquaculture to support local food security and employment and thereby decrease illegal activities. Part of these investments will go into multispecies hatcheries across the country and into Community Fish Landing Centers (CFLCs) in strategic areas. The rise of digital channels for transactions and delivery services^3^,^4^ can already be observed today.

In Malaysia, the spread of COVID-19 among the undocumented communities is having serious repercussions on public health and well-being. This has prompted actions by the authorities to better acknowledge and regulate their presence. There is an opportunity for the current legal framework to be revised and improved taking into account the health and well-being of the undocumented communities. Furthermore, the educational information about COVID-19 conveyed to the coastal communities contributes to the increased awareness of the importance of practicing hygienic lifestyles. It is foreseen that there will be a remarkably improved access to basic facilities including education and healthcare for the whole population, including the undocumented communities. We also expect stable internet and access to reliable sources of information across the country, including remote coastal communities in 10 years’ time.

In Vietnam, people came back to their traditional skills and livelihoods and realized the role of less tourists and natural systems. Local governments and residents have started to identify more sustainable livelihoods and improve their own resilience to cope with risks like a pandemic or climate change. Thus, the community learned to appreciate their less crowded island and recognize their own responsibilities in nature conservation. In 10 years’ time, the Cham islands are likely to become a national brand in the context of community-based ecotourism, limiting for the number of tourists, protecting marine biodiversity, and engaging the community to say no to plastic pollution.

In Indonesia, the communities living on the small island of Selayar have been forced to rely on subsistence agriculture activities to support themselves, if only temporarily. Nevertheless, the new agricultural skills obtained by fishers during this time of hardship may be beneficial to increase their resilience as it diversifies their source of nutrition and, potentially, income. It has to be acknowledged, however, that the environmental pressure on terrestrial ecosystems in small islands may not be able to support such shifts from coastal to terrestrial economic activities. In 10 years’ time, should the reliance on the terrestrial ecosystem to provide daily sustenance to the local communities persist, land-use changes brought about by human activities may alter the landscape of the small island. Previously pristine tropical forests will be changed to farmyards, which will, inevitably, cause lasting damages to the ecological integrity of the islands terrestrial ecosystems. The migration away from the island will likely increase, risking the demographic profile on the island to be dominated by an aging population.

In the United Kingdom, there has been a move to working online (or a blend of online and face-to-face delivery) for the provision of education and many office-based employees. Some people have learned new technological skills, or started up new businesses from home, which they will be able to use in the future. In addition, regional businesses in the North Devon Biosphere Reserve have been able to benefit from a rise in national tourists and an increased awareness of local produce since the supply chain for some products was affected by the pandemic. In 10 years’ time, the trend toward reduced commuting and more tourism from UK residents is likely to be maintained, as practices started during the pandemic are reinforced by the need to reduce the carbon emissions associated with travel. Residents who have discovered local outdoor spaces are likely to continue to visit these on a regular basis, increasing the footfall and visitor numbers in the natural environment.

### Opportunities Arising From COVID-19 Scenarios

The abrupt interruption of business as usual around the world provides an opportunity for change. For the development of future management plans for sustainability, the scenarios created for this paper provide valuable insights and areas that can serve as springboards. These potential springboards differ between locations and situations and therefore need to be individually identified.

For sustainable marine management, the appropriateness of locations for marine protected areas should be reviewed, considering different local and national food security and conservation needs with and after the COVID-19 pandemic. The consequences of the suspension or reduction in fishing activities should be documented and used to identify species that benefit from seasonal pauses in fishing. Information like this can be used to feed into sustainable marine resource management in the future. Sources of plastic litter and other pollutants are changing rapidly, causing new long-term risks. This will need to be addressed, politically and on a behavioral level, especially in regard to the increasing amount of single-use plastics and personal protective equipment being littered (Silva et al., 2021). Human interactions have been undergoing strict restrictions, some of which will remain in place for the foreseeable future such as social distancing and the usage of face covering. Under these conditions of extended times of social isolation, easily accessible and inclusive measures to support human health and well-being are becoming more relevant. It already shows that people’s mental, physical, and social health and well-being is suffering significantly, and further action is required (Pan et al., 2014). These are just a few examples of how abrupt interruptions identified in this paper can feed into long-term management solutions and thereby mitigate newly arising risk factors as well as utilize newly arising opportunities.

#### Limitations and Concerns

Since the COVID-19 situation has been uncertain and continuously changing, the discussions that we had during the annual meeting in July 2020 might not be completely timely anymore. Furthermore, no comprehensive empirical research was conducted to investigate the situation following the COVID-19 pandemic in all study sites. The scenarios are therefore not an objective representation of the current situation. Instead, they are a product of timely expert knowledge, experiences, and perceptions provided by local partners, researchers, and stakeholders. Despite all scenarios being built around the principles of sustainable development, each area has different priorities due to individual, biological, social, and political circumstances.

## Conclusion

The pandemic has provided a moment to stop, think, and potentially reset current practices. It is possible to make use of this window of opportunity and “build back better” (OECD, 2020). COVID-19 can lead society and individuals into a gloomy future, which is shaped by increasing amounts of poverty, desperate over-exploitation of ecosystems in order to survive as well as social distance, plastic waste, and poor physical and mental health. Our future scenario exploration suggests that COVID-19 can also enable a future that is characterized by independent small communities, less carbon emissions, newly announced nature reserves, sustainable and local harvest, and strong family bonds. Most opportunities described in this work are observed and reflected at the community level. On the governmental level, potential change might include reprioritization of existing policies that emphasize biodiversity recovery and the connectedness of humans with nature; it might include facilitating the diversification of livelihood options such as creating *additional* livelihoods rather than *alternative* livelihoods. Change might be reflected in a newly created dialogue beyond national boundaries with regard to human rights and migration. It will be key to find a sustainable balance between bringing income to the local communities again and protecting fragile ecosystems that just started to recover; this could potentially be accomplished with a future focus on slow tourism and the introduction of more systematic rest periods for fishing (Conway and Timms, 2010; Kroner et al., 2021).

## Data Availability Statement

The original contributions presented in the study are included in the article/Supplementary Material, further inquiries can be directed to the corresponding author/s.

## Author Contributions

IR conceptualized the manuscript idea, developed the outline, and led the workshop. MA was leading the GCRF Blue Communities project and supervises all collaborations. All other authors contributed equally to the co-creation of this final version of this manuscript in a highly collaborative effort. All authors contributed to the article and approved the submitted version.

## Conflict of Interest

The authors declare that the research was conducted in the absence of any commercial or financial relationships that could be construed as a potential conflict of interest.

## Footnotes

1 www.blue-communities.org

2 https://sdgs.un.org/goals

3 https://www.bfar.da.gov.ph/

4 http://pdp.neda.gov.ph/
