# Supplementary material for: Looking Through the COVID-19 Window of Opportunity: Future Scenarios Arising From the COVID-19 Pandemic Across Five Case Study Sites
Source: Front Psychol. 2021 Jul 7;12:635686. doi: 10.3389/fpsyg.2021.635686 (PMC8293277; doi:10.3389/fpsyg.2021.635686)
Supplement: Supplementary file 1 [file Table_1.DOCX]

| Selayar, Indonesia | | | |
| --- | --- | --- | --- |
| SDGs | **BAU** | **COVID-19** | **Opportunities** |
| Environment  Animals & wildlife  Biodiversity  Ecosystem Services  Valuation of nature  Habitats  Mangroves  Corals  Seagrass | Plenty of risks due to human activities, focused on getting subsistence. | Reduced risks, mainly for inland ecosystems, due to constraints on human activities. However, there may be more risks towards coastal environment, as most of them will have to resort to gleaning to obtain food | Increased quality for mainland ecosystems, such as forests and rivers. |
| Society  Community  Health & Wellbeing  Tradition & Culture  Diseases  Education  Youth  Social Roles  Urbanisation | Gloomy forecasts. Alcoholism & divorces are predicted to not be rare. | Gloomier forecasts. Children not being able to go to school would increase the pressure on parents in keeping watch & educating them. This could potentially increase the tension inside marriages. | None |
| Economy  Fisheries  Aquaculture  Illegal Activities  Drugs  Blast fishing  Tourism  Jobs and Opportunities  Alternative Livelihood  Seaweed  Global Relations  Money  Laws and implementation | Illegal activities are rampant due to perverse incentive (a quick way to get rich). Tourism is so so. | Illegal activities are reduced due to the decreasing market for their products.  There will also be lack of tourists which will reduce the opportunity to create alternative livelihoods. | Better ecosystem quality due to reduced destructive practices. |

| Cu Lao Cham, Vietnam | | | |
| --- | --- | --- | --- |
| SDGs | **BAU** | **COVID-19** | **Opportunities** |
| Environment  Animals & wildlife  Biodiversity  Ecosystem Services  Valuation of nature  Habitats  Mangroves  Corals  Seagrass | Highly exploited  Degradation  More on tourism  Increase value of nature  Habitats: polluted, worse  Mangroves reduce  Corals develop  Seagrass reduces  More polluted | more diverse wildlife, corals develop, beach and water cleaner; reduce pollution;  good for the environment. | Increase environment health (diverse wildlife, sand and water cleaner; fishing pressure decreases; decrease pollution) |
| Society  Community  Health & Wellbeing  Tradition & Culture  Diseases  Education  Youth  Social Roles  Urbanisation | Community: more diverse social stratification (more benefit difference, labours, income)  Health & wellbeing: more mental health issues (stress, conflict, aggressive…)  Tradition & culture: change in behaviour i.e. noisier; affected by foreign culture  Diseases: more diseases, related to environmental pollution, infected diseases  Education: same  Youth: more active, engaged  Social roles: same  Urbanisation: no | Community worrier due to the disease, health problem, and economic decrease;  Family/neighbour relationship closer.  Education: online learning/teaching | Community members’ relationship closer, more harmonious; promote traditional culture  Local people enjoy nature, peaceful island atmosphere; resilience.  Less pressure on earning, increase other values (family/ neighbour relationship) |
| Economy  Fisheries  Aquaculture  Illegal Activities  Drugs  Blast fishing  Tourism  Jobs and Opportunities  Alternative Livelihood  Seaweed  Global Relations  Money  Laws and implementation | Fishery: fish catch decrease; less fishermen  Aquaculture: no  Illegal activities: reduce  Drugs: no  Blast fishing: no  Tourism: increases, more people involve  Alternative livelihood: diverse, tend to tourism  Seaweed: little  Global relations: broaden,  Money: increase income, especially from tourism  Law and implementation: protecting nature; develop infrastructure, healthcare | Fishing catch decreases; fishing pressure decrease, tourism decreases; lower income;  Government support more (food, health care…) for the local community; develop seafood market (internal) | Traditional livelihoods increases, more sustainable (fishing, net making, forest leaf collecting).  Government supports more; develops domestic fish market |

| **Tun Mustapha Park, Malaysia** | | | |
| --- | --- | --- | --- |
| **SDGs** | **Business As Usual** | **COVID-19** | **Opportunities** |
| **Environment**  Animals & wildlife  Biodiversity  Ecosystem Services  Valuation of nature  Habitats  Mangroves  Corals  Seagrass | 1. Decline in fish and other wild marine fishery stock forced fishers to seek for alternative jobs.  2. Risk of marine species extinction (protected species, marine resource for human consumption and tourism) and polluted sea water (increase water borne diseases caused by improper waste management including human waste).  3. Increased coastal erosion led to increased loss of mangrove forest cover (functioned as a source of livelihood and buffer zone), thus risking the livelihood and life of people living in coastal areas.  4. Destruction of marine habitat threatened by destructive fishing activities such as fish bombing and use of sodium cyanide. This led to the decrease in fish population.  5. Reduced aesthetic quality of the island landscape (less forest cover and greenery) due to improved access and mobility of people.  6. Less wild sea cucumber to collect due to overfishing.  7. Increased pollution of coastal areas due to improper disposal of solid waste degrades the human living environment.  8. Conversion of forest cover to plantation degrades the soil quality, increases the runoff of water, use of fertilizer pollutes the land and water, and it reduces biodiversity. | Pollution  Reduced in noise pollution due to the Movement Control Order (MCO), which restricted mining, construction and boating activities (under water noise reduced).  [Negative outcome] Observed more Personal Protective Equipment (PPE) face masks as solid waste in the marine area.  No changes to destructive practices like deforestation although demand during MCO decreased.  Less fishing during COVID-19 in the beginning of MCO because it was unclear if fishing was still allowed or not (misinterpretation)  Less tourism reduces pressure on food chains and gives more room and quiet time to animals | New laws to regulate noise pollution.  New practices to regulate solid waste pollution are being discussed. (there is high need and it is visible how masks are being discarded everywhere)  Provides more time for fish stocks to recover  Less fishing which involves fish bombing leads to less damage to corals in the beginning of MCO.  Increased appreciation of nature for well-being, people see how spending time in nature makes them feel good  Opportunities to strengthen the measures to protect threatened marine species |
| **Society**  Community  Health & Wellbeing  Tradition & Culture  Diseases  Education  Youth  Social Roles  Urbanisation | Health, wellbeing & diseases: Increased drug addiction and sexually transmitted diseases (STDs) due to prostitution, low awareness on STDs (it is also considered a taboo topic, and human trafficking).  Communities are at higher risk of mental health problems.  Diseases: Poor hygiene practice among communities leading to the increase in disease transmission.  Diseases: Transmission of diseases through undocumented/unregulated immigration.  Youth: Drug abuse increased among youths in TMP.  Education & employment: Limited education opportunities for undocumented children which leads to limited access to formal employment further exacerbating poverty issues. Some fishers would turn to illegal poaching activities to sustain their livelihoods.  Community: Increased migration from rural to urban areas  Fish bombing continues leading to safety issues for recreational activities among the locals or other fishers or divers  Community: Issues of undocumented people/stateless persist. As the number of undocumented people grows, local Malaysian kids might grow up and marry undocumented people (female) which leads to their future children not receiving proper documentation (endless cycle).  Urbanisation: Labour exploitation (cheap labour, unsafe working environment and low wages) especially involving undocumented people.  Political issues: immigration especially in terms of undocumented people/stateless remains an issue in TMP. Government agencies often lack resources and some of the government servants engage in corrupt practices especially when dealing with undocumented people/stateless. | Health & wellbeing: Lockdowns and movement control orders (MCOs) during the pandemic which affects the communities’ livelihoods might further increase the risk of mental health problems.  Increase of mental stress due to limited movement freedom and less trade option to receive food or other goods.  Increased usage of telecommunication devices to log into social media and for entertainment purposes replacing the face-to-face interaction and to reduce boredom. This also leads to depression and mental illness  Diseases: Transmission of COVID-19 among undocumented people/stateless communities.  Education & employment: Undocumented people/stateless will face more difficulties in accessing formal services such as schooling, employment, etc. because immigration gets more difficult with the government being busy dealing with COVID  A wide range of invalidated information was circulated throughout the communities, creating confusion on what can and cannot be done during the MCO and what helps/what doesn’t against COVID. | The transmission of COVID-19 among undocumented people/stateless communities could pressure for political will to address the ongoing issue of undocumented/stateless and to create legal frameworks for easy immigration and give people a better life (eg.: offering visas such as IMM.13)  The local communities are motivated to cultivate agricultural crops on vacant lands available.  COVID-19 pandemic increases the local communities’ awareness towards the importance of hygiene thus prompting them to adopt a more hygienic lifestyle to prevent the spread of the virus.  The local communities become more skilled at using telecommunication devices and IT (tech-savvy).  Government agencies to create awareness on the importance of referring to official and reliable sources for information and latest updates. |
| **Economy**  Fisheries  Aquaculture  Illegal Activities  Drugs  Blast fishing  Tourism  Jobs and Opportunities  Alternative Livelihood  Seaweed  Global Relations  Money  Laws and implementation | Increased alternative livelihood options: BnB, full time tourist guides  Illegal activities- Surveillance in the park increase leads to more arrests  Fishing bombing leads to less tourists coming due to the fear of safety.  Constant worry about access to health service and education, no equal opportunities to employment without proper identity documentation. Abuse on the undocumented employees.  Mismatch of tourist’s expectation and experience due to lack of formal training of guides and language barrier. Tourists often misbehave and violate local norms because they are not informed properly  Decrease of seafood harvest due to high risk of fisheries extinction.  More forest is converted into oil palm plantations. | International tourism affected by Covid 19 as there were/are movement restrictions.  Lack of awareness and loose observation of protective rules and measures for COVID due to poverty (affordability of masks etc) and misinformation.  The requirement to comply to COVID-19 SOP in transporting products to major towns pose a hassle to the communities.  Sharply reduced demand for restaurants, hotels and shops which customers were majority international tourists.  Fisherman cannot sell catches due to low demand | Local tourism increased due to restriction to travel overseas and the demand of staycation and leisure activities increased (revenge tourism).  Focus on strengthening local tourism especially on developing ecotourism as a potential segment of domestic tourism to be promoted in TMP.  Opportunities to focus on business development in waste management and recycling.  Shift the focus on diversifying the local economic sectors and livelihood options among the coastal communities to avoid depending on one source of income.  Local communities are becoming more tech-savvy and able to explore online SMEs.  Unsold catches decompose and become fertilizers. |

| Palawan, the Philippines | | | |
| --- | --- | --- | --- |
| SDGs | **BAU** | **COVID-19** | **Opportunities** |
| Environment  Animals & wildlife  Biodiversity  Ecosystem Services  Valuation of nature  Habitats  Mangroves  Corals  Seagrass | More fishing | Less fishing  Vegetable backyard will be developed | More time for fish to recover/recruit |
| Society  Community  Health & Wellbeing  Tradition & Culture  Diseases  Education  Youth  Social Roles  Urbanisation | Frequent gossip with neighbour, and drinking alcoholic beverages  Healthy children  Frequent gambling (cockfighting and card games)  Children attend schooling physically  Different tv shows | Physical gossiping and drinking with neighbour greatly minimized  Children’s health impaired  Less gambling  Children stays home for schooling  Less tv shows | Social media platforms might further increase in demand  Online gambling might be patronized more  Digital telecommunications might increase in demand  Video blogging might replaced traditional tv shows |
| Economy  Fisheries  Aquaculture  Illegal Activities  Drugs  Blast fishing  Tourism  Jobs and Opportunities  Alternative Livelihood  Seaweed  Global Relations  Money  Laws and implementation | More fish catch  Mini store | Export of high valued fish will be reduced  Mini store might expand/close | Online business might bloom |

| North Devon Biosphere Reserve, UK | | | |
| --- | --- | --- | --- |
| BAU | **COVID-19** | **Opportunities** | **BAU** |
| Economy  Fisheries  Aquaculture  Illegal Activities  Drugs  Blast fishing  Tourism  Jobs and Opportunities  Alternative Livelihood  Seaweed  Global Relations  Money  Laws and implementation | They live in a small town in rural Southwest England, where Joe is a seaweed farmer, working near their home, and Mary works in IT, travelling to work in a nearby city.  They have a mortgage on the house they live in but are secure and comfortable money-wise.  Joe sells samphire to local restaurants and seaweed as speciality food; he also sells seaweed to the cosmetics industry.Joe has a loan to support his small business. | Mary could face redundancies in her company; she could be at risk herself.  Even if she can stay in her job, this would cause additional workload and stress because fewer colleagues (see health and well-being category).  It would be possible for her to work at home though, so she can continue to work during lockdown.  They could face a substantial loss of income, which would make it difficult to keep up mortgage and business loan payments and might put them into other debt in the medium term.  With no tourists in the area the market for Joe’s product is reduced. | Joe might think of other markets and products, perhaps more online ordering and postal delivery. He will rethink market and processing, which will give him new skills and business opportunities  IT mum Mary might decide to reduce her working hours to reduce stress (if she stays in work) – but that option would reduce the family income further; perhaps the solution is that she goes into Joe’s business and they expand. |
| Environment  Animals & wildlife  Biodiversity  Ecosystem Services  Valuation of nature  Habitats  Mangroves  Corals  Seagrass | As a family they are quite interested in sustainability and try to make efforts to be pro-environmental.  This was partly the reason for Joe starting the seaweed farm as a typical Southwest tradition but also sustainable business.  Living in a rural area they need to use a car quite a lot. Mary commutes to work by car every day. | Economic pressures may mean that politicians are less willing to give attention and funding to environmental improvement. | Better growth for the seaweed, cleaner water so better product  d  Mary is working from home and so she is driving much less, leading to improved air quality in the city.  There are many fewer tourists visiting so less pressure on systems such as water treatment. |
| Society  Community  Health & Wellbeing  Tradition & Culture  Diseases  Education  Youth  Social Roles  Urbanisation | The children like their schools on the whole and have good friends there, but Paul has experienced some conflict at his secondary school.  Many after-school activities and clubs such as music, sports, and they all like computer games | The kids will miss school and their friends and will feel more lonely and disconnected from their peer groups.  They will miss out on educational content, and this will particularly affect the younger ones. Their development and education might be delayed. Paul may be anxious because he is approaching end-of-school exams. | For Paul, educational content will be delivered online, and he will learn additional IT skills.  Paul might have better well-being because he is no longer exposed to the conflicts at school.  All children will potentially learn more practical skills by spending more time with their dad; he might take them out to work with him, where it is safe to do so. |
|  | The family like their holidays and usually travel quite a bit within the UK to see family, and also abroad, especially Spain where they rent a house by the sea.  They also get out in local nature and visit cafés as a family.  They live in an area that is attractive to tourists and has considerable tourism all year round. | Local restaurants and hotels struggle with the lower tourist numbers; demand for seaweed products drops.  The family miss seeing their relatives, although have family Zoom quizzes to keep in touch. | When lockdown eases, people are reluctant to travel abroad and many more UK people take holidays in the southwest UK.  People are flying much less, reducing carbon emissions. |
|  | They are all healthy in general and have a healthy diet and some exercise. | Working at home would pose additional strain and conflict for Mary and Joe, because they have to juggle childcare with work.  This is likely to impact more on Mary because she would be at her desk working remotely, whereas Joe would working outside the house.  Relationships with other friends and family might become more distanced. | Grandparents might move in with them to help with the childcare, which could reduce the stress (but pose other conflicts and trigger space issues).  The extended family including grandparents might get closer to each other and build much stronger relationships that will last into the future and build a foundation especially for the children |
